# Workmen's Contributions

**Published:** 1909-11-27

**Authors:** W. Pearson

**Affiliations:** Secretary


### Editor's Letter-Box.

### WORKMEN'S CONTRIBUTIONS.

*To the Editor of* The Hospital.

Dear Sir,—As another instance of the working men's appreciation of hospitals, may I point out that only last week a committee, composed of working men drawn from the various workshops of the town and district, handed to the chairman of this hospital a cheque for £106 8s. 10d., being the proceeds of a parade and carnival held in October; the idea conceived, the movement started and organised entirely by working men. Nor is this all the working men do for this hospital. By means of weekly collections in the works and other places of industry, over £400 annually is contributed to our funds. This, together with the amount realised by the carnival, about represents one-seventh part of our income, and entirely provided by the resources of the labouring classes. Yours faithfully,

W. Pearson, Secretary.

